# Effects of accreditation on United States and Canadian veterinary college libraries in the nineteenth and twentieth centuries

**DOI:** 10.5195/jmla.2020.882

**Published:** 2020-04-01

**Authors:** Susanne K. Whitaker, Vicki F. Croft

**Affiliations:** Retired Veterinary Medical Librarian, Flower-Sprecher Veterinary Library, Cornell University, Ithaca, NY, skw2@cornell.edu; Retired Head of the Animal Health Library, Washington State University College of Veterinary Medicine, Pullman, WA, croft@wsu.edu

## Abstract

**Objective:**

The authors’ objective was to document the effects of evolving veterinary accreditation standards on the development of currently existing accredited US and Canadian veterinary school libraries in the late nineteenth and twentieth centuries.

**Methods:**

We gathered historical standards that major veterinary accreditation agencies developed with respect to libraries and library services. Historical background on college libraries, their facilities, services, and personnel via surveys, literature searches, and archival documents was also collected. We then correlated the evolving standards with each library’s development.

**Results:**

There was a marked correlation between the prevailing accreditation standards and library development, particularly during the post–World War II era and through the mid-1980s. These impacts—which included new and separate facilities, hiring of professional librarians, and additional open hours—affected not only the twenty new developing veterinary schools, but also the libraries of the preexisting colleges.

**Conclusions:**

Professional veterinary accrediting standards were an important influence on the evolution of veterinary school libraries, particularly during the years of major growth in the number of new veterinary colleges in the United States and Canada. In the 1990s and beyond, both libraries and accreditation standards continue to evolve in response to changes in technology, economics, publishing methods, and more. This latter is a story yet to be told.

## INTRODUCTION

Although the first veterinary colleges in the United States and Canada were established in the late 1800s, it was not until the early twentieth century that veterinary college libraries and their services and collections developed. Cornell University (1897), Colorado State University (1907), University of Guelph (1909), Iowa State University (1912), and University of Pennsylvania (1913) were early leaders of this movement. This was followed by periods of growth and maturation of other libraries and collections throughout the remainder of the century. Many factors contributed to library development, but one common denominator was the influence of changes in accreditation agency recommendations. This paper focuses on the impacts of these recommendations on the libraries serving the thirty-two currently accredited veterinary schools in the United States and Canada that were established in the nineteenth and twentieth centuries.

## METHODS

A search of the literature of accreditation agencies involved in developing standards for veterinary schools was conducted, and sections relating to libraries, librarians, and services were compiled. This history of the recommendations and their content that were established by the US Bureau of Animal Industry (BAI) and continued by the US Veterinary Medical Association (USVMA) [renamed the American Veterinary Medical Association (AVMA) in 1898] was researched (supplemental Appendix A). In addition, a table including the founding dates of the current veterinary colleges, veterinary libraries, and reading rooms as well as the dates of the first professional librarians was compiled (supplemental Appendix B). Two offshore schools in the Caribbean, Ross University and St. George’s University, and veterinary schools established after 2000 were excluded. Correlations between library development and standards were identified and analyzed.

## RESULTS

### American Veterinary Medical Association approval and accreditation

In 1863, during the third year of the Civil War, the USVMA was founded not only to elevate the veterinary art in this country, but also to remove “the nation’s veterinary profession from the grasp of uneducated men” [1]. Prior to this time, the few veterinary surgeons, as they were then known in the United States, were uneducated or, at best, self-educated: “Those interested in practicing veterinary medicine trained themselves as best they could from the scant literature available” or apprenticed to a practitioner [2]. The small number of early trained veterinarians, most of whom had been educated in Europe, was insufficient to meet the needs of growing animal epizootic diseases. Also, during the Civil War, the US Army was reluctant to hire unqualified veterinary surgeons to care for the huge number of horses involved in the conflict: “Civil War–era veterinary schools had been primitive, lacking in trained educators, and primarily [were] located in livery stables or similar structures in large cities” [1]. “The profession’s early leaders clearly recognized the need [for improvements in veterinary medicine] and were influential in convincing legislators and the public that scientific veterinary institutions were needed” [1].

“Conceived with a commitment to advance and promote veterinary education, the United States Veterinary Medical Association moved to achieve these aims by [almost immediately] forming the Committee on Intelligence and Education (CIE) in 1863” [2], one of the USVMA’s first standing committees. The committee remains the association’s longest continuously existing body, one that would later become the AVMA’s Council on Education. Due to its concern about the quality of veterinary medical education, the AVMA passed a resolution in 1871 calling for the New York College of Veterinary Surgeons to administer an entrance examination to “further the object of a higher grade of education” for veterinarians [2]. The USVMA’s first attempt to persuade veterinary colleges to improve the quality of education had no effect as the new organization had no leverage to induce reforms.

As noted in historical publications, “As of 1900, the number of schools of veterinary medicine in the United States totaled 16, a half of which number were privately owned, and the remainder [five] were the operating academic departments of land-grant agricultural colleges” [3, 4]. As the result of the first Morrill Land-Grant Act signed by President Abraham Lincoln in 1862, a number of state universities established colleges focusing on agriculture and the mechanical arts, some of which began offering courses in veterinary science. Later in 1890, Congress passed a second Morrill Act that provided an annual appropriation to support the land-grant colleges. As time went on, many of those early veterinary courses evolved into organized colleges of veterinary medicine awarding the doctor of veterinary medicine (DVM) degree. They formed the foundation for present day veterinary medical education in this country.

On the other hand, from the end of the Civil War until the turn of the century, a few private or proprietary colleges had been founded in urban environments primarily to fulfill the need to care for working horses. However, literally “no standardization of education existed between the schools, and quality remained haphazard and irregular” [2]. Those private, proprietary, non-land-grant, non-university affiliated schools were excluded from this library-related study because all were closed by 1927, as noted in the list of “Former Veterinary Institutions in the United States” [5, 6].

Interestingly, those private schools had opened without regard for the USVMA’s membership qualification requirement. Effective on January 1, 1893, it stated that candidates should be graduates of veterinary schools that offered a minimum three-year curriculum taught by at least four faculty. This bold move was a forerunner of accreditation standards to provide leverage aimed toward educational reform. However, no mention was made as to how a school would be “recognized” [1–3].

When the veterinary school at Harvard University closed in 1901, the CIE stepped up its efforts to improve veterinary education beyond simply scrutinizing school catalogs and studying replies to survey form letters. In expanding its leadership role in 1905, the CIE reported at the AVMA’s forty-second annual meeting in Cleveland on the results of an extensive thirty-four-question survey letter to gather information about veterinary colleges [3, 7]. Subsequent to that survey, the CIE took steps in 1906 to initiate a national college evaluation program, conducted largely by questionnaire. As reported at the 1906 convention, an approved list had been developed of “veterinary colleges…endorsed by the Association…as qualifying graduates for eligibility to membership in the Association” [3]. All of the colleges of veterinary medicine in the United States and Canada were notified during the next two years that the AVMA would undertake a classification of the colleges considering their curriculum, faculty, and physical equipment [8]. Under the heading “Material Equipment,” it was stated that “There must also be a well-equipped library and each of the departments will need to have its own museum collection” [9].

After several years of wrangling exacerbated by strict government reforms, the AVMA instituted a four-year curriculum requirement beginning with the 1916/17 academic year. Four years of high school became a prerequisite in 1920. Still lacking sufficient leverage for enforcement, a CIE recommendation was approved, empowering it to outline what might be regarded as requirements for acceptable veterinary colleges. This included a schedule whereby veterinary colleges might be granted accreditation. In doing so, plans of the Council on Medical Education of the American Medical Association (AMA) were closely studied to develop an evaluation system based on curriculum, faculty, and physical equipment [10]. The AMA established its Council on Medical Education and Hospitals (CMEH) in 1904, adopted early “ideal standards” for medical schools in 1905, and began a series of inspections in 1906, resulting in the first classification of schools a year later [11]. Of the ten categories of qualifications on which the AMA based its rankings, the last specified “libraries, museum, charts and teaching equipment.”

When the CMEH released its findings in 1907, it exposed deficiencies and initiated a “great wave” of improvement in human medical education across the country. Findings from the first survey were never published, but with support from the Carnegie Foundation, results of the second survey were issued in 1910 in what became known as “the Flexner Report.” This influential report also presented the state of library facilities at each institution at that time [12].

At the fifty-eighth annual AVMA meeting held in Denver on September 5–9, 1921, the committee’s report was presented, resulting in adoption of the first detailed version of “Essentials of an Approved Veterinary School” [10]. These essentials were a forerunner of the current Committee on Education (COE) standards. The AVMA CIE also anticipated conducting periodic site visits of schools, but such inspections were rare [2].

In 1928, an amendment to the AVMA Bylaws terminated the CIE. A new COE was created in its place with additional members. The COE was charged with publishing a list of AVMA-recognized schools, along with on-site inspections. All were visited by at least one committee member in the 1930s [2]. The quality of education continued to improve as schools implemented higher requirements.

In 1946, the entire structure of the AVMA was reorganized. A further expanded COE with increased membership was formed to replace the earlier COE. In cooperation with the Canadian Veterinary Medical Association (CVMA), a CVMA representative was granted full member voting status and participated in site accreditation visits, with greater presence for Canadian institutions [2, 13]. The “Essentials of an Approved Veterinary School” were revised, and a three-level school classification system was established granting full approval, probational approval, or disapproval [2].

Today, the AVMA COE “is the body that makes accreditation decisions based upon the *Standards for Accreditation*.” Moreover, since 1949, the COE has also been recognized by the Council on Higher Education Accreditation and its predecessors as the accrediting body for veterinary medicine [14]. The COE has conducted inspections of schools at least once every seven years as part of the AVMA accreditation program. It should also be noted that in 1973, the AVMA began extending the approval process to apply to international schools outside the United States and Canada [15].

### US Department of Agriculture Bureau of Animal Industry and US Civil Service Commission accreditation

In the 1880s, Congress passed the Animal Industry Act, which was signed into law by President Chester A. Arthur in May 1884, creating the BAI as a division of the US Department of Agriculture (USDA). Importantly, its chief officer was to be a veterinarian. Primarily intended to prevent diseased animals from being used as food, BAI work involved promoting livestock disease research through the eradication of the most damaging and most communicable livestock diseases, such as Texas tick fever and bovine pleuropneumonia. The bureau enforced animal import regulations and controlled the interstate movement of animals. The BAI was also interested in educational standards, especially after the Meat Inspection Act of 1906 became law. In 1908, a BAI proposal to upgrade veterinary education received approval from the US Civil Service Commission to enable examination and hiring of the hundreds of professionally qualified veterinarians who were needed as meat inspectors.

In spring of 1908, the secretary of agriculture appointed a select committee, and schools in the United States and Canada were visited to further expand on-site inspection efforts. The resulting *BAI Circular 133* [16], dated and approved in June 1908, contained rankings and recommendations on faculties, equipment, teaching, and minimum course instruction. The eighteen schools that were visited were ranked into three classes: A (acceptable), B (unacceptable), or C (new institutions not yet having graduated a class or unacceptable). Only graduates from class A schools could sit for the Civil Service Examination required for BAI employment [2, 17].

That same year, the BAI began supervising veterinary schools. The 1908 report had specified twenty-seven recommendations for how veterinary colleges might operate. “For a school to remain on the bureau’s acceptable list, it had to comply with a series of prescriptive recommendations” that were so strict that any deviation, including changing textbooks, required permission. Nevertheless, many schools began implementing the government’s regulations. The AVMA CIE raised concerns due to the stringent nature and rigidity of the curriculum and faculty teaching specifications [2].

While there was some opposition to grading or classifying schools, “the American Veterinary Medical Association in convention in 1908 generally endorsed the US Secretary of Agriculture’s approved report for the long-term beneficial efforts that it might exert on the nation’s veterinary medical educational system” [3]. When rewritten a year later, the nineteen regulations that were presented in *BAI Circular 150* [17], dated September 1909, served as criteria that the BAI used to evaluate educational institutions for many years. When also adopted by the AVMA upon resumption of the school evaluation program in 1912, the regulations became a reference standard in the association’s bylaws a year later [3].

In 1918, the BAI was directed to supervise the educational programs of the veterinary colleges. Their influence was such that “In 1921, the BAI employed more than 1,500 full-time veterinarians, about 10 percent of the profession” as federal inspectors and agents, and “One of the requirements for employment was graduation from a bureau-acceptable veterinary school” [2].

The BAI supervisory program continued until it was relinquished to the AVMA. In 1956, the National Commission on Accreditation recognized the AVMA as the official accrediting association for veterinarians, but it was not until 1961 that the Civil Services Commission concurred, followed by the US Army for staffing its Veterinary Corps in 1966 [18].

### Library requirements

Although these early “standards” dealt extensively with the curriculum, buildings, admission credentials, and faculty qualifications, little mention was made of libraries. Nevertheless, in 1906, AVMA CIE requirements for a national evaluation program of “standards” contained a section under “Material Equipment.” It stated that “There must also be a well-equipped library and each of the departments will need to have its own museum collection” [9].

Regrettably, neither *BAI Circular 133* [16], “Report and Recommendations Regarding Veterinary Colleges in the United States” (1908), nor *BAI Circular 150* [l7], “Regulations Governing Entrance to the Veterinary Inspector Examination” (1909), addressed textbooks, libraries, or collections. The lack of recognition for information resources in these BAI circulars was ironic because by the early twentieth century, some land-grant universities were already beginning to develop central and/or departmental libraries on their campuses and building collections, catalogues, and services that supported student instruction in various subject areas. Schools offering veterinary courses would have been acquiring holdings of contemporary and standard veterinary books and periodicals [19].

In the text of the 1921 AVMA “Essentials for an Approved Veterinary College,” libraries were included under “General Teaching Facilities and Instruction”:

e) The college should have a working library, to include the more modern veterinary text and reference books together with related scientific text-books and journal files. The library should receive regularly the leading veterinary and related scientific periodicals, the current numbers of which should be in racks or on tables easily accessible to the students. At the end of each year these periodicals should be bound and added to the files of bound periodicals. The library room should be properly lighted and heated, and open during all or the greater part of the day. [10]

By 1941, twelve AVMA-accredited schools had been evaluated using the first set of 1921 standards. Libraries were included under “Physical Plant” in that version of the “Essentials of an Acceptable Veterinary School:

4. The school should enjoy the use of modern buildings sufficient in size to provide lecture rooms, class laboratories, small laboratories for members of the teaching staff and advanced students, administrative offices, and a medical library…A trained librarian should be employed to supervise the operation and development of the library and to keep it open and available for student use for not less than six hours per day for not less than five days per week. The library should receive the leading veterinary journals, at least a few of the medical journals and the more important journals dealing especially with anatomy, physiology, bacteriology, parasitology and pathology. The current numbers of these publications should be on racks or tables readily accessible to students and the completed volumes should be bound and [kept] equally available to students. [20]

There was little change until 1956, when the library merited its own standards section and some of the specifics were eliminated, making the overall requirements more general:

II. Essential Requirements

5) Library. Adequate library facilitates are essential to a sound program of veterinary medical education and research. The library should be established as a part of the veterinary medical school; it should be well housed, conveniently located, and available for the use of students and faculty at all reasonable hours. It should be administered by a professionally trained or experienced librarian and should be adequately sustained both for operation and for the purchase of current periodicals and other accessories of veterinary medical importance. [21]

At intervals over approximately the next fifty years, standards for libraries continued to be revised to incorporate additional components, such as library and information resources and facilities, and generally became more well defined. For instance, in 1980, the section name was changed to “Library and Learning Resources.” A statement was also added about libraries being appropriately staffed. The purchase of learning resources was also specified [22]. In 1985, wording related to providing public services and supporting continuing education was added to essential library service functions [23].

Then, in 1993, a phrase was inserted in the library standards concerning the “adequate collection of appropriate instructional and research materials for each subject, including books, periodicals, specimens, and non-print media, supplemented with electronic reference materials and retrieval systems” [24]. In 2001, it was deemed necessary to add “timely access” in providing library resources and services [25].

More recent changes have emphasized electronic access to and retrieval of information, while de-emphasizing the physical aspects and changing a qualified librarian to a “human resource.” The standards for libraries in use as of 2017 read:

Standard 5, Information Resources

Timely access to information resources and information professionals must be available to students and faculty at core training sites. The college shall have access to the human, digital, and physical resources for retrieval of relevant veterinary and supporting literature and development of instructional materials, and provide appropriate training for students and faculty. The program must be able to demonstrate, using its outcomes assessment data, that students are competent in retrieving, evaluating, and efficiently applying information through the use of electronic and other appropriate information technologies. [26]

### Effect on libraries

The founding of US and Canadian veterinary schools can be grouped into four historical periods, as adapted from Smith et al. [27, 28]: the legacy colleges (1862–1920) [group I], twelve schools; post–World War II colleges (1944–1963) [group II], nine schools; the 1970s colleges (1968–1983) [group III], ten schools; and twenty-first century colleges (1998–2014) [group IV], five schools (supplemental Appendix B).

While Cornell University, University of Guelph, University of Pennsylvania, Iowa State University, Colorado State University, and Ohio State University were early leaders in creating reading rooms and libraries or hiring librarians [29], the other group I schools that had been established between 1862 and 1920—notably Auburn University, Washington State University, Texas A&M University, and Kansas State University—have documented compliance regarding one or more of the above prior to the mid-1950s, when the new 1956 standards were adopted.

The new standards had a major influence on the nine group II schools that began operation between 1944 and 1963. All eventually established standalone veterinary libraries or reading rooms. The seven pre-1956 schools were all in the process of moving in that direction, and some had already hired professional librarians. About half of group II schools were hiring library staff about the same time the schools were established. This was a new trend.

For the ten group III schools (1968–1983), professional librarians (except one paraprofessional) were hired at or close to the founding of the respective veterinary school. In at least two cases (Virginia Tech and Louisiana State University), preplanning for library services and staff began well before the veterinary college was established. Interestingly, even if the standards regarding location of veterinary collections or libraries had not changed, only seven had separate veterinary libraries or reading rooms. The others were part of a campus-wide, agricultural, or medical school library.

None of the five group IV (1998–2014) libraries established separate veterinary libraries. All provided the services of professional librarians housed in the main campus library, and none were full-time veterinary librarians.

## DISCUSSION

According to a 1930 US Office of Education survey of land-grant colleges and universities [19], “The libraries of land-grant institutions are to a large extent a development of the twentieth century.” Many of the eastern schools had “assembled by 1890 collections of books fairly adequate for the period,” while others “had not had the same opportunity to obtain similar foundations for their libraries.” Some were noted to have initiated substantial development by 1920, while others had not increased book and periodical expenditures for many years. Moreover, rather than a librarian or designated staff person, responsibility for small libraries was typically “entrusted to some professor who gave the library such attention as he could spare from his regular duties,” which affected its character and use. Nevertheless, “before 1900 the needs for a full-time professional librarian and increased purchases of books were being strongly urged in many institutions. These rumblings presaged the development that took place in the first quarter of the twentieth century” [19].

Concurrent with a growing recognition of the importance of libraries to instruction for land-grant institutions in general, this trend was also beginning to be implemented to support veterinary education. Most of the early veterinary colleges were located on campuses of land-grant universities (supplemental Appendix B).

Although the “Essentials” that were first adopted in 1921 specified a “working library,” it was not until 1941 that the first official requirements for libraries became a part of the AVMA standards [20]. Even though libraries were lumped together with classrooms and laboratories, most schools did have either a reading room or, in some cases, a separate library.

In 1956, libraries were finally given full recognition with their own separate standards section that featured the word “essential.” Part of the text called for a “trained professionally or experienced librarian; adequately sustained for operations and purchase of current periodicals” [21]. It also stated that libraries should be conveniently located as part of the veterinary school. That section noted the importance of libraries and librarians to the veterinary college accreditation process.

It was at this point that colleges started getting serious about library standards. In fact, less than twenty years later, Kerker would note the progress by remarking that, in 1959, there were only five professional librarians in the United States and Canada, while by 1977, there were twenty-five, a fourfold increase [30]. Several librarians from this era remarked in oral histories that they were hired to meet the 1956 standards for a professional librarian [31–33]. The same can be surmised for other colleges. By 1979, all group I and II colleges had separate libraries or reading rooms (University of Florida had a main medical library that supported a small reading room).

Accreditation teams, as well as veterinary school administrators, were well aware of the AVMA library requirements. This awareness is documented by accounts from two legacy colleges and one post–World War II college in quotes taken from archival records of Washington State College, one of the legacy schools. A 1948 internal accreditation team report read: “The Committee was disappointed to find that their recommendations made in the 1946 inspection as related to the library have been disregarded or overlooked” [34]. A “librarian is needed,” and “a library [should] be built up as rapidly as possible.” The veterinary administration was so concerned that, in 1959, the college conducted a survey of library facilities in other colleges of veterinary medicine [35]. However, it was not until the 1965 accreditation site visit report that they were “commended for establishing their new veterinary library.”

Five years later, Kansas State University, another established group I veterinary school, did a similar survey of library facilities and practices [36]. That institution subsequently constructed a new, enlarged veterinary library, which opened in 1970 with its first professional veterinary librarian (supplemental Appendix B). Thirdly, in 1949, accreditors for the Tuskegee University School of Veterinary Medicine, a post–World War II college, recommended that a reading room should be established in one of the veterinary buildings. As a result, in the early 1950s, a library committee was appointed and a small unstaffed reading room was established in the Clinical Anatomy Building, but it was not until 1967 that a professional librarian was hired. Subsequently, a new and expanded library facility became part of the school’s Learning Resource Center in 1979 [37].

Between 1960 and 1985, eleven new veterinary colleges were established. In light of the expanded 1956 standards, seven of the ten new group III schools began moving toward adding new library facilities and all hired professional librarians. Furthermore, several of the group I or II schools opened libraries or reading rooms, and several more employed full time and/or professional veterinary librarians. This was a very active time for growth and development of US and Canadian veterinary libraries, staffing, and services.

The next change in standards came in 1980 when audiovisual materials, referred to as “autotutorials” at that time, were added to library accreditation standards, recognizing diversity of learning technologies beyond printed books and journals. The 1980 revision was renamed “Library and Learning Resources.” The library section was largely unchanged, but an information resources paragraph was added.

In 1993, the AVMA library standards section added electronic materials and access along with information retrieval. This was the beginning of a move away from simply counting physical collection volumes or holdings and noting the extent of physical facilities, such as library seating capacities. Parent university libraries were beginning to recognize the need to provide expanded liaison or outreach services, so it was not unusual that, in following years, no new veterinary library facilities were established. The offices of those veterinary liaison librarians were often located off-site in main or other campus libraries, while still being readily available for providing information support for veterinary college faculty and students and in continuing to build animal health collections.

Given changes in campus administrative structures and technology, twenty-first-century colleges have been covered under less restrictive requirements regarding location, facilities, and personnel for library support, while instead mandating additional remote and electronic access to resources.

## CONCLUSION

The development of US and Canadian veterinary school libraries as physical entities in the nineteenth and twentieth centuries was dynamic and influenced by a number of factors. Accreditation standards, originally established by the US BAI and later assumed by the AVMA, were a factor that had a significant impact on the development of library facilities, the hiring of professional librarians, and the services offered for the twentieth century. A correlation between the date that the veterinary school was established and the standards in place at that time was found, particularly in the post–World War II colleges. Moreover, the new standards also had an influence on the older more established schools.

After these libraries reached a pinnacle of recognition in the 1970s and 1980s, factors apparent in the last twenty years are rapidly precipitating numerous changes in many of them. These include actually closing branch and unit campus libraries; downsizing collections and space with reliance on off-site storage facilities; moving from print to electronic scholarly resources; changing educational technologies to favor computer-based modalities in teaching veterinary medicine; introducing new services and technologies—such as 3D printing, coffee services, laptop checkouts, and food—in the library policies; expanding outreach services, including faculty research and scholarly collaborations; and incorporating social events, such as wine and cheese receptions and exhibits. All of these changes affect the way users perceive the library as a physical and intellectual resource in general. Due to economics, downsizing, changes in publishing, and consolidation, it is not surprising that separate veterinary libraries are no longer in vogue.

Changes in AVMA COE accreditation standards are responding to and driving many of these changes along with economics and technology. A subsequent history of veterinary libraries and collections in the twenty-first century will be much different from the past and is a story yet to be written.

## SUPPLEMENTAL FILES

Appendix A American Veterinary Medical Association (AVMA) veterinary college accreditation standards related to libraries, 1907–2017 (excludes USDA Bureau of Animal Industry requirements)

Appendix B Establishment of veterinary colleges and veterinary libraries in the United States and Canada, 1862–2014

## 

**Susanne K. Whitaker, AHIP**, skw2@cornell.edu, Retired Veterinary Medical Librarian, Flower-Sprecher Veterinary Library, Cornell University, Ithaca, NY

**Vicki F. Croft, AHIP, FMLA**, croft@wsu.edu, Retired Head of the Animal Health Library, Washington State University College of Veterinary Medicine, Pullman, WA
