# Supplementary material for: Effects of accreditation on United States and Canadian veterinary college libraries in the nineteenth and twentieth centuries
Source: J Med Libr Assoc. 2020 Apr 1;108(2):167–76. doi: 10.5195/jmla.2020.882 (PMC7069832; doi:10.5195/jmla.2020.882)
Supplement: Appendix A [file jmla-108-167-s001.pdf]

JMLA Journal of the Medical Library Association

SC
Supplemental content to
J Med Libr Assoc. Apr;108(2):dx.doi.org/10.5195/jmla.2020.882
www.jmla.mlanet.org
© Whitaker, Croft 2020

Effects of accreditation on United States and Canadian veterinary college libraries in the nineteenth and twentieth centuries

Susanne K. Whitaker, AHIP; Vicki F. Croft, AHIP, FMLA

APPENDIX A

American Veterinary Medical Association (AVMA) veterinary college accreditation standards related to libraries, 1907–2017 (excludes USDA Bureau of Animal Industry requirements)

1907

[Under Material Equipment:] "There must also be a well-equipped library and each of the departments will need to have its own museum collection."

> Source: Pearson L. Veterinary colleges: report of the Committee on Intelligence and Education of the American Veterinary Medical Association. Am Vet Rev. 1907 Oct;32:16–32.

1921/1922

e) The college should have a working library, to include the more modern veterinary text and reference books together with related scientific text-books and journal files. The library should receive regularly the leading veterinary and related scientific periodicals, the current numbers of which should be in racks or on tables easily accessible to the students. At the end of each year these periodicals should be bound and added to the files of bound periodicals. The library room should be properly lighted and heated, and open during all or the greater part of the day.

> Source: Way C, Day LE, Goss LW, Simms BT, Bergman HD; Committee on Intelligence and Education. Essentials of an approved veterinary college. J Am Vet Med Assoc. 1922 Jan;LXns;13(4):495. (Available from: <https://hdl.handle.net/2027/uc1.b3300345>. [cited 15 Sep 2019].).

1941

III. Physical Plant: 4. The school should enjoy the use of modern buildings sufficient in size to provide lecture rooms, class laboratories, small laboratories for members of the teaching staff and advanced students, administrative offices, and a medical library…A trained librarian should be employed to supervise the operation and development of the library and to keep it open and available for student use for not less than six hours per day for not less than five days per week. The library should receive the leading veterinary journals, at least a few of the medical journals and the more important journals dealing especially with anatomy, physiology, bacteriology, parasitology and pathology. The current numbers of these publications should be on racks or tables readily accessible to students and the completed volumes should be bound and keep equally available to students.

> Source: American Veterinary Medical Association. Essentials of an acceptable veterinary school (approved 1941). 1945 AVMA directory. Chicago, IL: The Association; 1945. p. 22.

1946

[Same wording, but library appears under Physical Plant No. 4:]

> Source: American Veterinary Medical Association. Essentials of an acceptable veterinary school (rev. December 1946). 1950 AVMA directory. Chicago, IL: The Association; 1950. p. 50.

SC
Supplemental content to
J Med Libr Assoc. Apr;108(2):dx.doi.org/10.5195/jmla.2020.882
www.jmla.mlanet.org
© Whitaker, Croft 2020

1956

[Separate section for libraries under II. Essential Requirements:]

5) Adequate library facilities are essential to a sound program of veterinary medical education and research. The library should be established as a part of the veterinary medical school; it should be well housed, conveniently located, and available for the use of students and faculty at all reasonable hours. It should be administered by a professionally trained or experienced librarian and should be adequately sustained both for operation and for the purchase of current periodicals and other accessories of veterinary medical importance.

> Source: American Veterinary Medical Association. Essentials of an acceptable veterinary medical school (rev. October 1956). 1962 AVMA directory. Chicago, IL: The Association; 1962. p. 82.

1973

[No change]

> Source: American Veterinary Medical Association. Essentials of an acceptable veterinary medical school. (rev. July 1973). 1974 AVMA directory. Chicago, IL: The Association; 1974. p. C-78.

1980

III. Essential Requirements.

5) Library and learning resources: Adequate library facilities are essential to a sound program of veterinary medical education and research. The library should be established as part of the college. It should be well housed, appropriately staffed, conveniently located, and available for the use of students and faculty at all reasonable hours. It should be administered by a professional[ly] trained or experience[d] librarian and should be adequately budgeted for both operation and the purchased of learning resources...

There shall be an adequate collection of learning resources for each subject: specimens, audio-visual materials, auxiliary apparatus, and animals for demonstration purposes.

> Source: American Veterinary Medical Association. Essentials of an acceptable veterinary medical school. (rev. July 1980). 1981 AVMA directory. Schaumburg, IL: The Association; 1981. p. 413.

1985

C. Essential Requirements:

5) Library and learning resources: A library is essential to veterinary medical education, research, public services, and continuing education. Library facilities must be adequately housed, appropriately staffed, conveniently located, and available for the use of veterinary students and faculty at all reasonable hours. The library should be administered by a qualified librarian and must be adequately budgeted for both operation and the purchase of leaning resources.

There shall be an adequate collection of appropriate instructional materials for each subject, including books, periodicals, specimens, and audio-visual materials.

> Source: American Veterinary Medical Association. Essential requirements of an accredited or approved college of veterinary medicine. (approved July 1985). 1986 AVMA directory. Schaumburg, IL: The Association; 1986. p. 567.

Supplemental content to
J Med Libr Assoc. Apr;108(2):dx.doi.org/10.5195/jmla.2020.882
www.jmla.mlanet.org
© Whitaker, Croft 2020

1990

[No change]

> Source: American Veterinary Medical Association. Essential requirements of an accredited or approved college of veterinary medicine. (rev. July 1990). 1991 AVMA directory. Schaumburg, IL: The Association; 1991. p. 617.

1993

5. [First paragraph remains the same:]

There shall be an adequate collection of appropriate instructional and research materials for each subject, including books, periodicals, specimens, and non-print media, supplemented with electronic reference materials and retrieval systems.

> Source: American Veterinary Medical Association, Council on Education Accreditation. II. Accreditation policy. B. Essential requirements of an accredited or approved college of veterinary medicine (rev. August 1993). 1994 AVMA directory and resource manual. Schaumburg, IL: The Association; 1994. p. 196.

2001

Standards

5) Library and Information Resources:

[First paragraph the same; "timely access" inserted after collection of in second paragraph.]

> Source: American Veterinary Medical Association. Essential requirements of an accredited or approved college of veterinary medicine. Standard requirements of an accredited or approved college of veterinary medicine. 2001 AVMA directory and resource manual. Schaumburg, IL: The Association; 2001. p. 209.

2003 (and remained in effect through 2010)

Standards

Library and Information Resources

Libraries and information retrieval are essential to veterinary medical education, research, public services, and continuing education. Timely access to information resources, whether through print, or electronic media or other means must be available to students and faculty. The library shall be administered by a qualified librarian.

> Source: American Veterinary Medical Association. Standard requirements of an accredited or approved college of veterinary medicine. 2003 AVMA directory and resource manual. Schaumburg, IL: The Association; 2003. p. 21.

2017

Standard 5, Information Resources:

Timely access to information resources and information professionals must be available to students and faculty at core training sites. The college shall have access to the human, digital, and physical resources for retrieval of relevant veterinary and supporting literature and development of instructional materials, and provide appropriate training for students and faculty. The program must be able to demonstrate,

JMLA Journal of the Medical Library Association

SC

Supplemental content to
J Med Libr Assoc. Apr;108(2):dx.doi.org/10.5195/jmla.2020.882
www.jmla.mlanet.org
© Whitaker, Croft 2020

using its outcomes assessment data, that students are competent in retrieving, evaluating, and efficiently applying information through the use of electronic and other appropriate information technologies.

Source: American Veterinary Medical Association. COE accreditation policies and procedures: requirements [Internet]. Schaumburg, IL: The Association; Sep 2017 [cited 15 Sep 2019]. <https://www.avma.org/ProfessionalDevelopment/Education/Accreditation/Colleges/Pages/coe-pp-requirements-of-accredited-college.aspx>.
