# Supplementary material for: Effects of accreditation on United States and Canadian veterinary college libraries in the nineteenth and twentieth centuries
Source: J Med Libr Assoc. 2020 Apr 1;108(2):167–76. doi: 10.5195/jmla.2020.882 (PMC7069832; doi:10.5195/jmla.2020.882)
Supplement: Appendix B [file jmla-108-167-s002.pdf]

JMLA Journal of the Medical Library Association

Supplemental content to
J Med Libr Assoc. Apr;108(2):dx.doi.org/10.5195/jmla.2020.882
www.jmla.mlanet.org
© Whitaker, Croft 2020

Effects of accreditation on United States and Canadian veterinary college libraries in the nineteenth and twentieth centuries

Susanne K. Whitaker, AHIP; Vicki F. Croft, AHIP, FMLA

APPENDIX B

Establishment of veterinary colleges and veterinary libraries in the United States and Canada, 1862–2014

Institution (current name)	State or prov -ince	Land grant instit-ution	Date school estab-lished*	First class matric-ulated*	Date first veterinary reading room	Date separate veterinary library†	Date first veterinary librarian	Date first professional veterinary librarian	Notes
**The legacy colleges (1862–1920)**					10 United States; 2 Canadian				
University of Guelph	ON		1862	1862	1909?	???	1909 (Sweetapple)	1975? (Hull)	Separate veterinary library closed in 2009.
Iowa State University	IA	Yes	1879	1880	1912	1976	1915 (Flemming)	1971 (Peterson)	Veterinary student library in 1943.
University of Pennsylvania	PA		1884	1884	??	1913	1912 (Kimball)	1953 (Cross)	Reading room prior to 1913.
Ohio State University	OH	Yes	1885	1887	1917	1929	1929 (Kramer)	1961 (Merhemic)	Veterinary Lab Library in 1917.
Cornell University	NY	Yes	1894	1896		1897	1897 (Cornell)	1946 (Purington)	Library founded by gift from New York State Governor Roswell P. Flower in 1897. Later renamed Flower-Sprecher Library in 1993.

JMLA Journal of the Medical Library Association

SC
Supplemental content to
J Med Libr Assoc. Apr;108(2):dx.doi.org/10.5195/jmla.2020.882
www.jmla.mlanet.org
© Whitaker, Croft 2020

Institution (current name)	State or prov -ince	Land grant instit-ution	Date school estab-lished*	First class matric-ulated*	Date first veterinary reading room	Date separate veterinary library†	Date first veterinary librarian	Date first professional veterinary librarian	Notes
Washington State University	WA	Yes	1899	1899	1952	1963	1964 (Gass)	1976 (Croft)	Library renamed Animal Health Library in 2009.
Kansas State University	KS	Yes	1905	1905	ca. 1937	1970	1970 (Coffee)	1970 (Coffee)	
Auburn University	AL	Yes	1907	1909	1935	1970	1970 (Henley)	1970 (Henley)	Named Charles A. Cary Veterinary Library in 1970.
Colorado State University	CO	Yes	1907	1907	1907	1979	1957 (Bergland)	1957 (Bergland)	Veterinary Teaching Hospital Library established 1979.
Michigan State University	MI	Yes	1907	1910	1960		1960 (Maher)	1960 (Maher)	The branch library closed in 2012 and became a reading room only.
Texas A&M University	TX	Yes	1916	1916		1949	1949 (Hicks)	1949 (Hicks)	Veterinary Library until it became Medical Sciences Library in 1978 serving several colleges.
Université de Montréal	QC		1920	1920			1971 (Jette)	1971 (Jette)	School founded in 1886; located at Oka from 1920 until it moved to St.-Hyacinthe in 1947.

JMLA Journal of the Medical Library Association

Supplemental content to
J Med Libr Assoc. Apr;108(2):dx.doi.org/10.5195/jmla.2020.882
www.jmla.mlanet.org
© Whitaker, Croft 2020

Institution (current name)	State or prov -ince	Land grant instit-ution	Date school estab-lished*	First class matric-ulated*	Date first veterinary reading room	Date separate veterinary library†	Date first veterinary librarian	Date first professional veterinary librarian	Notes
**Post–World War II colleges (1944–1963)**					8 United States; 1 Canadian				
University of Illinois	IL	Yes	1944	1948	1947	1952	1952 (Estep)	1952 (Estep)	Moved to new veterinary school campus in 1982.
Tuskegee University	AL	Yes	1945	1945	Early 1950s; 1967 (staffed)	1979	1967 (Davis)	1967 (Davis)	Unstaffed reading room created in early 1950s. Named T.S. Williams Veterinary Medical Library in 1979.
University of California–Davis	CA	Yes	1946	1948	1946	1956	1959 (Merala)	1959 (Merala)	Carlson Health Sciences Library serves as veterinary library.
University of Georgia	GA	Yes	1946	1946	1956	1968	1968 (Milton)	1980 (Rowland)	School opened, then closed, then reopened. Current reading room opened in 1968.
University of Missouri	MO	Yes	1946	1946	1951	1970	1951 (Hombs)	1970 (Boyd)	Moved to current location 1977. Renamed Zalk Veterinary Medical Library in 2005.
University of Minnesota	MN	Yes	1947	1947	1948	1971	1948 (Rukavina)	1956 (Raynolds)	Moved to current location 1971.

JMLA Journal of the Medical Library Association

SC
Supplemental content to
J Med Libr Assoc. Apr;108(2):dx.doi.org/10.5195/jmla.2020.882
www.jmla.mlanet.org
© Whitaker, Croft 2020

Institution (current name)	State or prov -ince	Land grant instit- ution	Date school estab- lished*	First class matric- ulated*	Date first veterinary reading room	Date separate veterinary library†	Date first veterinary librarian	Date first professional veterinary librarian	Notes
Oklahoma State University	OK	Yes	1948	1948		1950	n.d. (Linscheid)	1967 (MacNeil)	Named William E. Brock Memorial Library in 1980.
Purdue University	IN	Yes	1957	1959		1959	1959 (Kerker)	1959 (Kerker)	Moved to new library in 1995.
University of Saskatchewan	SK		1963	1965		1965	1965 (Martin)	1965 (Martin)	Had previous “library” in 1940s.
**The 1970s colleges (1968–1983)**					9 United States; 1 Canadian				
Louisiana State University	LA	Yes	1968	1973		1974	1974 (Loubiere)	1974 (Loubiere)	M. Hanchey began planning for library in 1969. Moved to current location in 1974.
University of Florida	FL	Yes	1971	1976			1966 (Srygley)	1966 (Srygley)	No separate veterinary library. Reading room now closed.
Mississippi State University	MS	Yes	1974	1977		1977	1977 (Hsuing)	1977 (Hsuing)	
University of Tennessee	TN	Yes	1974	1976		1977	1977 (West)	1977 (West)	Pendergrass Agriculture-Veterinary Medicine Library established 1977, serving several colleges.
North Carolina State University	NC	Yes	1975	1981		1980	1979 (Fischer)	1979 (Fischer)	Named William Rand Kenan, Jr. Library in 2006.

JMLA Journal of the Medical Library Association

SC
Supplemental content to
J Med Libr Assoc. Apr;108(2):dx.doi.org/10.5195/jmla.2020.882
www.jmla.mlanet.org
© Whitaker, Croft 2020

Institution (current name)	State or prov -ince	Land grant instit- ution	Date school estab- lished*	First class matric- ulated*	Date first veterinary reading room	Date separate veterinary library†	Date first veterinary librarian	Date first professional veterinary librarian	Notes
Oregon State University	OR	Yes	1975	1979		1979	1979 (Bates)	2005 (Christie)	
Tufts University	MA		1978	1979		1978	1978 (Inglis)	1978 (Inglis)	Named Webster Family Library.
Virginia Tech/ University of Maryland	VA	Yes	1978	1980	1978	1980	1980 (MacNeil)	1980 (MacNeil)	V. Kok began building collection in 1975.
University of Wisconsin–Madison	WI	Yes	1979	1983			1983 (Sessions)	1983 (Sessions)	No separate veterinary library.
University of Prince Edward Island	PI		1983	1986		1986	1986 (Taylor)	1986 (Taylor)	No separate veterinary library.
**Twenty-first century colleges (1998–2014)**					4 United States; 1 Canadian				
Western University of Health Sciences	CA		1998	2003			1978 (Stevenson)	1978 (Stevenson)	Main library established 1978. No separate veterinary library.
University of Calgary	AB		2005	2008			2006 (Toews)	2006 (Toews)	No separate veterinary library.
Utah State University	UT	Yes	2009	2012			2008 (Weingart)	2008 (Weingart)	No separate veterinary library.

JMLA Journal of the Medical Library Association

SC
Supplemental content to
J Med Libr Assoc. Apr;108(2):dx.doi.org/10.5195/jmla.2020.882
www.jmla.mlanet.org
© Whitaker, Croft 2020

Institution (current name)	State or prov -ince	Land grant instit- ution	Date school estab- lished*	First class matric- ulated*	Date first veterinary reading room	Date separate veterinary library†	Date first veterinary librarian	Date first professional veterinary librarian	Notes
Midwestern University	AZ		2012	2014			2011 (Reed)	2011 (Reed)	No separate veterinary library.
Lincoln Memorial University	TN		2014	2014			2012 (Sharrow)	2012 (Sharrow)	No separate veterinary library.

* Official date the veterinary college was founded (not the parent institution) with year of that college's first matriculating (graduating) doctor of veterinary medicine (DVM) class.

Source: Smith DF, Isham GK, ed. Pathways to progress: the vision and impact of members of the Association of American Veterinary Medical Colleges at the fiftieth anniversary [1966–2016]. Washington, DC: Association of American Veterinary Medical Colleges; 2016.

† At some institutions, a separate veterinary or animal health library was created; at others, a life sciences or medical library may contain materials dedicated to supporting the veterinary school as noted.

Sources: Various sources were used to compile data concerning the veterinary libraries, including personal communications from current and past veterinary medical librarians (2015 and 2019), published and unpublished veterinary library histories, American Veterinary Medical Association (AVMA) directory and resource manuals, and other resource materials. Copies of personal communications are available in the MLA Veterinary Medical Libraries Section Archives located at the Medical Sciences Library, Texas A & M University, College Station, TX.
